# External Validation Study of First Trimester Obstetric Prediction Models (Expect Study I): Research Protocol and Population Characteristics

**DOI:** 10.2196/resprot.7837

**Published:** 2017-10-26

**Authors:** Linda Jacqueline Elisabeth Meertens, Hubertina CJ Scheepers, Raymond G De Vries, Carmen D Dirksen, Irene Korstjens, Antonius LM Mulder, Marianne J Nieuwenhuijze, Jan G Nijhuis, Marc EA Spaanderman, Luc JM Smits

**Affiliations:** ^1^ Care and Public Health Research Institute (CAPHRI) Department of Epidemiology Maastricht University Maastricht Netherlands; ^2^ School for Oncology and Developmental Biology (GROW) Department of Obstetrics and Gynecology Maastricht University Medical Centre Maastricht Netherlands; ^3^ Center for Bioethics and Social Sciences in Medicine University of Michigan Medical School Ann Arbor, MI United States; ^4^ Care and Public Health Research Institute (CAPHRI) Maastricht University Maastricht Netherlands; ^5^ Research Centre for Midwifery Science Faculty of Health Zuyd University Maastricht Netherlands; ^6^ Care and Public Health Research Institute (CAPHRI) Department of Clinical Epidemiology and Medical Technology Assessment (KEMTA) Maastricht University Medical Centre Maastricht Netherlands; ^7^ School for Oncology and Developmental Biology (GROW) Department of Pediatrics Maastricht University Medical Centre Maastricht Netherlands

**Keywords:** external validation, first trimester, prediction models, pregnancy, risk assessment

## Abstract

**Background:**

A number of first-trimester prediction models addressing important obstetric outcomes have been published. However, most models have not been externally validated. External validation is essential before implementing a prediction model in clinical practice.

**Objective:**

The objective of this paper is to describe the design of a study to externally validate existing first trimester obstetric prediction models, based upon maternal characteristics and standard measurements (eg, blood pressure), for the risk of pre-eclampsia (PE), gestational diabetes mellitus (GDM), spontaneous preterm birth (PTB), small-for-gestational-age (SGA) infants, and large-for-gestational-age (LGA) infants among Dutch pregnant women (Expect Study I). The results of a pilot study on the feasibility and acceptability of the recruitment process and the comprehensibility of the Pregnancy Questionnaire 1 are also reported.

**Methods:**

A multicenter prospective cohort study was performed in The Netherlands between July 1, 2013 and December 31, 2015. First trimester obstetric prediction models were systematically selected from the literature. Predictor variables were measured by the Web-based Pregnancy Questionnaire 1 and pregnancy outcomes were established using the Postpartum Questionnaire 1 and medical records. Information about maternal health-related quality of life, costs, and satisfaction with Dutch obstetric care was collected from a subsample of women. A pilot study was carried out before the official start of inclusion. External validity of the models will be evaluated by assessing discrimination and calibration.

**Results:**

Based on the pilot study, minor improvements were made to the recruitment process and online Pregnancy Questionnaire 1. The validation cohort consists of 2614 women. Data analysis of the external validation study is in progress.

**Conclusions:**

This study will offer insight into the generalizability of existing, non-invasive first trimester prediction models for various obstetric outcomes in a Dutch obstetric population. An impact study for the evaluation of the best obstetric prediction models in the Dutch setting with respect to their effect on clinical outcomes, costs, and quality of life—Expect Study II—is being planned.

**Trial Registration:**

Netherlands Trial Registry (NTR): NTR4143; http://www.trialregister.nl/trialreg/admin/rctview.asp?TC=4143 (Archived by WebCite at http://www.webcitation.org/6t8ijtpd9)

## Introduction

Perinatal mortality is an important quality indicator of perinatal care. The main causes of perinatal mortality are asphyxia, preterm birth (PTB), and born small-for-gestational-age (SGA) [1,2]. Pre-eclampsia (PE) is commonly related to SGA and induced preterm birth [3]. Another concern is the rising incidence of gestational diabetes mellitus (GDM), leading to large-for-gestational-age (LGA) infants [4]. Children born LGA are at increased risk of asphyxia and birth injuries [5]. Early identification of pregnancies at risk of these complications is important considering the substantial short- and long-term consequences for the health of mother and child. Women at high risk could benefit from further testing, increased surveillance, and preventive interventions.

A number of first trimester prediction models have been published addressing important obstetric outcomes including PE, GDM, spontaneous PTB, and infants born SGA or LGA [6]. These risk models are based on maternal characteristics, routine antenatal tests (eg, blood pressure), and sometimes include more complex predictors like specialized tests (eg, uterine artery Doppler and cervix length measurements) or biomarkers. Although some complex factors have been reported to improve discrimination, a drawback is that most of these tests provide additional costs, are not readily available in general antenatal settings, and are possibly inconvenient for pregnant women [7].

While the reported performance of most non-invasive prediction models is promising [7], few models have been externally validated in independent cohorts [8-16]. Evaluating the model’s performance in another population than the one used for model development is crucial before applying a model in daily practice to guide patient care [17,18].

This paper describes the design of a study aimed to externally validate existing first trimester obstetric prediction models, based upon maternal characteristics and standard measurements (eg, blood pressure), for the risk of PE, GDM, spontaneous PTB, and SGA and LGA infants among Dutch pregnant women (Expect Study I). Results of a pilot study on the feasibility and acceptability of the recruitment process and the comprehensibility of the Pregnancy Questionnaire 1 are also reported. Adequately performing models will be considered for use in clinical practice. We are planning an impact study—Expect Study II—to evaluate the application of adequately performing models (in association with tailored care paths) as compared with care-as-usual in Dutch obstetric care.

The specific objectives of the Expect Study I are (1) to identify published first trimester obstetric prediction models, based solely upon maternal characteristics and standard measurements (eg, blood pressure), for the outcomes PE, GDM, spontaneous PTB, SGA infants, and LGA infants; (2) to evaluate prospectively the predictive performance of these first trimester obstetric prediction models in a Dutch cohort of pregnant women; (3) to update, if necessary, the best performing models to the validation cohort; and (4) to measure maternal health-related quality of life, costs, and satisfaction aspects of current Dutch obstetric care for use as care-as-usual comparison to the intended Expect Study II.

## Methods

### Selection of Prediction Models

Systematic searches were performed in PubMed to identify all published first trimester obstetric prediction models using “prediction model” and its synonyms as search terms combined with relevant outcome terms and MeSH terms. The search terms were restricted to title and abstract fields (tiab). The detailed search strategies are available in Multimedia Appendix 1. Articles written in languages other than English, German, French, or Dutch were excluded. Citation lists of relevant articles were checked to select additional articles. The search was first performed in April 2013, before finalizing the study questionnaires, and will be updated before the start of each validation analysis per outcome. The first author screened all citations, and together with the last author, assessed the eligibility of the full text articles. In cases of disagreement, a third reviewer was used.

Prediction models were eligible for consideration if the following criteria were met: (1) the article presented the development of a prediction model or an update of a previously developed model, (2) the model contained multiple predictors, (3) predictors were routinely collected in Dutch obstetric care (maternal characteristics or blood pressure), (4) predictors were available and/or measured before 16 weeks and 0 days of gestation, (5) the model was based on weighted risk predictors, and (6) outcome of the model was PE, GDM, spontaneous PTB, SGA infants, or LGA infants. Authors of the original studies were contacted if the model intercept, regression coefficients, or definitions of predictors were not available.

### Study Design and Population

A multicentre prospective cohort study was performed among women living in the south-eastern part of The Netherlands (province of Limburg). Six hospitals and 36 midwifery practices recruited pregnant women less than 16 weeks pregnant and aged 18 years or older between July 1, 2013 and January 1, 2015. Follow-up took place until December 31, 2015. Pregnancies ending in a miscarriage, termination before 24 weeks of gestation, and women lost-to-follow-up were excluded.

The Medical Ethics Committee (MEC) of the Maastricht University Medical Centre evaluated the study protocol and declared that the study did not fall within the scope of the Dutch Medical Research Involving Human Subjects Act (WMO) (MEC 13-4-053). An independent physician was available for consultation by (eligible) participants.

### Recruitment

Eligible pregnant women visiting their midwife (approximately 85%) or obstetrician (approximately 15%) in the first trimester of pregnancy received verbal and written information about Expect Study I [19]. They were also asked whether they were willing to receive further information by email or telephone. If so, contact details were entered into an online system by their caregiver and used to send an automated information email about the study. Pregnant women were asked to complete a Web-based questionnaire before 16 weeks of gestation (Pregnancy Questionnaire 1) and 6 weeks after the due date (Postpartum Questionnaire 1). During the visit, blood pressure and heart rate were routinely measured and the results were given in writing to the women on the information leaflet in order to self-report in Pregnancy Questionnaire 1 [20,21].

Study questionnaires could be accessed through the Expect Study website [22] by use of a personal login code contained in the written information and information email. Women agreeing to participate gave online informed consent and answered the eligibility criteria before the start of Pregnancy Questionnaire 1. Paper-and-pencil questionnaires were available upon request. Three reminders were sent by email during 3-day intervals if Pregnancy Questionnaire 1 was not accessed or incomplete. Women who completed Pregnancy Questionnaire 1 were invited 6 weeks after the due date to complete Postpartum Questionnaire 1. Three email reminders were sent during 6-day intervals, and in case of non-response, a paper-and-pencil version of Postpartum Questionnaire 1 was sent (provided that the postal address was available). In Pregnancy Questionnaire 1, women were invited to fill out, on an optional basis, 3 additional questionnaires about costs, quality of life, and satisfaction of current obstetric care around 24 and 34 weeks of gestation (Pregnancy Questionnaires 2 and 3), and 6 weeks after the due date together with Postpartum Questionnaire 1 (Postpartum Questionnaire 2). Again, automatic reminders were sent out in case of non-response. Pregnancy status was asked at the beginning of Pregnancy Questionnaires 2 and 3. Women who reported a miscarriage or termination were referred to the end of the questionnaire and not invited for further questionnaires. Women not responding to Pregnancy Questionnaire 2 received no further invitations for the additional questionnaires, only for Postpartum Questionnaire 1. Women not responding to Pregnancy Questionnaire 3 were invited; however, for Postpartum Questionnaire 2. Medical records and discharge letters were requested from care providers.

A pilot study was carried out in the region of Maastricht before the official start of inclusion (March 25, 2013 to May 10, 2013) to assess the feasibility and acceptability of the recruitment process and the comprehensibility of Pregnancy Questionnaire 1. Evaluation questions about the recruitment process and form, content, and clarity of the questions were added to Pregnancy Questionnaire 1. If permission was given, participants were also approached by telephone.

### Data Collection

Inclusion, follow-up, and data collection of participants were managed by use of a logistic application specifically developed for Expect Study I. Questionnaires were developed by the research team and where possible, validated questionnaires were included.

Pregnancy Questionnaire 1 contained questions about the following topics: socio-demographic characteristics, anthropometric data, medical conditions, obstetric history, lifestyle, medication, vitamin and mineral supplements, fruit intake, dietary intake of vitamin D and calcium (selection questions from the Dutch National Food Frequency Questionnaire tool [23]), sun exposure, family history of medical conditions and obstetric outcomes, mental health (Edinburgh Depression Scale [24,25]), health status (EQ-5D-3L and cognitive dimension [26,27]), current pregnancy, and blood pressure and heart rate measurements.

The following aspects were collected in Postpartum Questionnaire 1: pregnancy outcome, pregnancy complications, labor and delivery, and neonatal outcomes. We also added several questions about the biological father.

The additional questionnaires—Pregnancy Questionnaires 2 and 3 and Postpartum Questionnaire 2—assessed maternal health status (EQ-5D-3L and cognitive dimension [26,27]), state anxiety (State-Trait Anxiety Inventory [28]), patient satisfaction, and costs of current obstetric care. Satisfaction was assessed antepartum (Pregnancy Questionnaires 2 and 3) by means of the Patient Satisfaction Questionnaire Short Form (PSQ-18) [29] and postpartum (Postpartum Questionnaire 2 or delivered at Pregnancy Questionnaire 2 or 3) by the Pregnancy and Childbirth Questionnaire (PCQ) [30]. To evaluate the costs of current obstetric care, all midwifery, hospital, and other care institution costs associated with care for pregnant women and their newborns from the beginning of pregnancy up to around 6 weeks after the due date were requested. In Pregnancy Questionnaire 3 and Postpartum Questionnaire 2, the date of the last completed additional questionnaire was indicated so that participants could see what period was to be covered.

Data from the medical records and letters of discharge were extracted and entered into a predesigned datasheet using Microsoft Access. All records were verified by a second researcher.

An overview of the items collected in the study questionnaires and data extracted from medical records and discharge letters is provided in Multimedia Appendix 2.

### Outcome Measures

Primary study outcomes were maternal and perinatal adverse outcomes predicted by the selected prediction models. The maternal outcomes were PE and GDM. PE was defined as pregnancy induced hypertension (PIH) accompanied by proteinuria (at least 300 mg protein in a 24 hour urine collection) [31]. PIH was defined as systolic blood pressure of at least 140 mmHg and/or diastolic blood pressure of at least 90 mmHg (Korotkoff V) after 20 weeks gestation, measured twice in a previously normotensive woman [31,32]. GDM was defined as a diagnosis of hyperglycemia during pregnancy, in a woman without pre-existing diabetes mellitus. The Dutch national guideline, in line with the World Health Organization guideline on Diagnosis and Classification of Diabetes Mellitus, defined hyperglycemia as the presence of either a fasting plasma glucose of 7.0 mmol/l or greater or 2-hour plasma glucose of 7.8 mmol/l or greater following a 75 g oral glucose tolerance test [33,34]. Perinatal outcomes included spontaneous PTB, SGA infants, and LGA infants. Spontaneous PTB is a delivery before 37 weeks of gestation started by primary contractions or spontaneous rupture of membranes. SGA and LGA were defined as an infant with a birth weight below the 10th percentile or above the 90th percentile, respectively, corrected for gestational age, ethnicity, gender, and parity [35].

The following secondary outcomes associated with the primary outcomes and important determinants of child morbidity and mortality were also measured: perinatal death (stillbirth or death within 7 days after birth, after 22 weeks of gestation), asphyxia (Apgar score of less than 7 after 5 minutes), admission to a neonatal intensive care unit (within 28 days after birth), SGA infants below the 2.3 percentile, PTB before 32 weeks of gestation, severe PE (delivery before the 34th completed week), instrumental delivery, cesarean section, and referral from midwife to obstetrician during delivery.

### Sample Size

No generally accepted rules are available for the calculation of required sample sizes for external validation studies of prediction models. We followed the rule of thumb by Vergouwe et al (2005), which states that at least 100 events and 100 non-events are necessary in order to be able to detect relevant differences between model performance in the derivation set and the validation set [36]. Assuming that each primary outcome would affect 4% or more of the pregnancies, we needed to collect data from about 2500 women. We aimed to recruit 2750 women, allowing for 10% loss to-follow-up.

### Statistical Analysis

Data analysis of the external validation study is in progress. Missing values will be handled by imputation, as analysis of only complete cases can lead to biased results [37]. Predictive performance of each prediction model will be evaluated by assessing discrimination and calibration [38,39]. Discrimination is the ability to distinguish between individuals who will develop the outcome from those who will not and will be assessed by calculating the c-index (area under the receiver operating characteristic curve [AUROC]). Calibration is the degree of agreement between predicted and observed probabilities. We will evaluate whether models may benefit from recalibration. Based on their final calibration and discriminative power, models will be ranked with respect to their predictive performance. The statistical analysis will be described in detail in the validation articles.

## Results

### Pilot Study

A total of 6 midwifery practices and 1 university hospital invited 95 pregnant women to participate. In total, 25 (26%, 25/95) women gave informed consent, of whom 21 (84%, 21/25) completed Pregnancy Questionnaire 1 fully and 4 (16%, 4/25) only partially because of technical problems. Of the participants, 70 (74%, 70/95) invited women who did not wish to fill out Pregnancy Questionnaire 1 could have return a non-participation form, but only 1 form was returned indicating that the woman “did not want to invest time in research.” The participants were positive about the recruitment process and only minor revisions were needed in the content of Pregnancy Questionnaire 1. In reaction to the low response rate, we made improvements to the recruitment process by asking contact details of informed pregnant women to send reminders about the study by email or telephone. Furthermore, a leaflet was designed to make the written information more concise and attractive, and we distributed information through social media and posters for promotion. Lastly, half of the pilot study participants declared that an incentive would increase their motivation to participate, and that they preferred higher probability of receiving a low-cost reward in comparison to a lower chance of getting an expensive incentive. On the basis of this information, low-to-medium cost incentives were invoked in the recruitment procedure (lottery of 27 gift cards and 2 photo shoots).

### Validation Cohort

**Figure 1:**
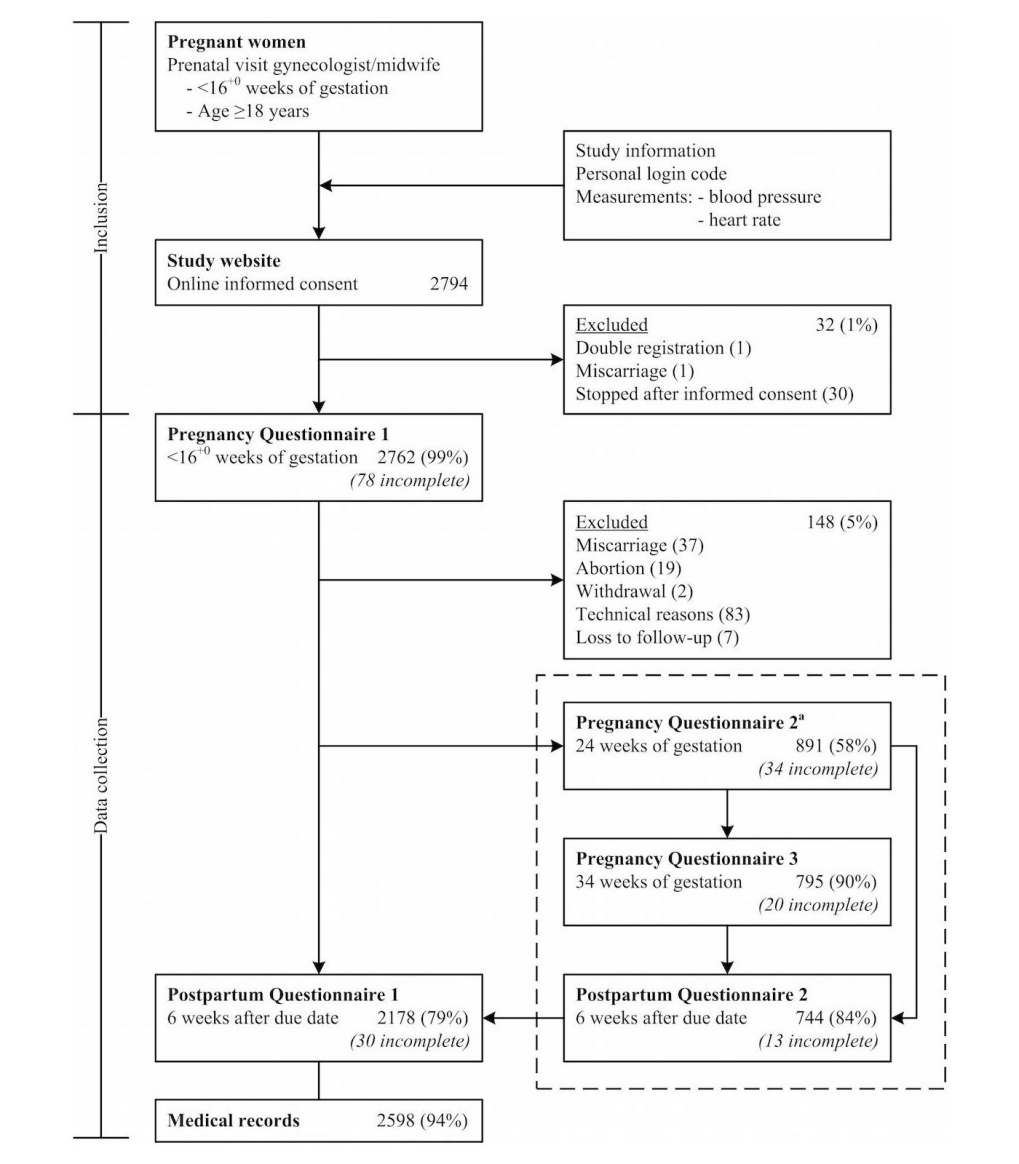
Inclusion and data collection of Expect Study I. The components in the dotted box represent the additional questionnaires. A total of 1548 participants gave permission to receive additional questionnaires (a).

The flowchart for enrolment and data collection of the validation cohort is shown in Figure 1. A total of 2794 women accessed the study website and gave online informed consent. Pregnancy Questionnaire 1 and Postpartum Questionnaire 1 were filled out by 2762 (98.85%, 2762/2794) and 2178 (78.86%, 2178/2762) women, respectively. Medical records were retrieved for 2598 (94.06%, 2598/2762) women. A completed Postpartum Questionnaire 1 or medical record was available for 2614 (94.64%, 2614/2762) women (validation cohort). General baseline characteristics and the primary outcomes of the validation cohort are shown in Table 1.

**Table 1:** Baseline characteristics and primary outcomes validation cohort of Expect Study I (N=2614).

Baseline characteristics (less than 16 weeks of gestation)		Observed validation cohort, n (%)
Age in years, mean (SD)		30.2 (3.9)
**Ethnicity**		
	Caucasian	2533 (96.90%)
	African-Caribbean	3 (0.11%)
	Asian	20 (0.77%)
	Hispanic	11 (0.42%)
	Mixed	47 (1.80%)
Tertiary education		1420 (54.32%)
**BMI^a^, mean (SD)**		24.2 (4.3)
	<18.5	87 (3.33%)
	18.5-24.9	1665 (63.70%)
	25-29.9	585 (22.38%)
	30-39.9	263 (10.06%)
	≥40	9 (0.34%)
**Medical history**		
	Chronic hypertension	28 (1.07%)
	Diabetes mellitus	11 (0.42%)
	Renal disease	5 (0.19%)
**Smoking during pregnancy**		
	Ever	318 (12.17%)
	Current	157 (6.01%)
**Alcohol consumption during pregnancy**		
	Ever	479 (18.32%)
	Current	9 (0.34%)
Nulliparous		1326 (50.73%)
**Conception**		
	Spontaneous	2440 (93.34%)
	Ovulation induction	93 (3.56%)
	IVF^b^/ICSI^c^	81 (3.10%)
**Obstetric history**		
	Prior pre-eclampsia	72 (2.75%)
	Prior gestational diabetes mellitus	15 (0.57%)
	Prior preterm birth <37 weeks of gestation	141 (5.39%)
	Prior birth weight <10th percentile	108 (4.13%)
	Prior birth weight >90th percentile	170 (6.50%)
**Primary outcomes**		
	Pre-eclampsia	76 (2.91%)
	Gestational diabetes mellitus	74 (2.83%)
	Spontaneous PTB <37 weeks of gestation	127 (4.86%)
	Birth weight <10th percentile	206 (7.88%)
	Birth weight >90th percentile	224 (8.57%)

^a^BMI: body mass index measured in kg/m^2^.

^b^IVF: in vitro fertilization.

^c^ICSI: intracytoplasmic sperm injection.

Of the women included in the validation cohort, 1548 (59.22%, 1548/2614) gave permission to be invited for the additional questionnaires. Pregnancy Questionnaire 2 was filled out by 891 (57.56%, 891/1548) women. Of the women who started the first additional Pregnancy Questionnaire 2 and were still pregnant, 795 (89.5%, 795/888) women filled out Pregnancy Questionnaire 3. Postpartum Questionnaire 2 was filled out by 744 (83.5%, 744/891) women.

## Discussion

Here, we describe the protocol of a study that aims to assess the predictive performance of multiple first trimester obstetric prediction models within an independent Dutch population. In this way, prediction models with similar outcomes can be compared and best performing models can be selected [40].

In the evaluation of a prediction model, external validation is an essential step. Generally, the predictive performance of the model decreases in the validation dataset due to model over-fitting in the development cohort [18,41]. Existing independent external validation studies of non-invasive, first trimester obstetric prediction models for GDM showed stable discriminative performances, with the highest AUROCs for the models by Nanda et al (AUROC 0.79) and Van Leeuwen et al (AUROC 0.76-0.77) [13-16]. For the outcomes early and late PE, only a few models based upon maternal characteristics and blood pressure have been externally validated and AUROCs declined to around 0.70 [8-12]. A limitation is that the numbers of events in these validation studies were (extremely) low, especially for early PE. No independent external validation studies of non-invasive prediction models for overall PE, spontaneous PTB, SGA infants, and LGA infants have been published.

The main strength of our study is the prospective cohort design, which enables optimal measurement of predictors and outcomes [42]. Recruitment by multiple centers improves the likelihood of obtaining a representative sample of the obstetric population, which is especially important in the obstetric care system in The Netherlands in which most pregnant women start antenatal care with a midwife. Web-based questionnaires were used as a data collection tool, which is efficient in a population with high access to the Internet, as it improves data quality and less missing data due to the incorporation of validation checks. Moreover, it is also more user-friendly in comparison to paper-pencil forms as non-relevant follow-up questions could be hidden, speeding up completion [43].

If one or more prediction models turns out to be externally valid, eventually after model updating, it is not self-evident that the model will be useful in clinical practice. The prediction models can only lead to improved outcomes for mother and child if they can guide healthcare professionals and individuals in their decision making regarding further management that are tailored to individual risk profiles, including additional testing, preventive interventions, lifestyle changes, monitoring, or treatment [42].

Statistical performance measures are important aspects of a prediction model, but they do not indicate its clinical usefulness. Even if the statistical performance is less good, the model may predict better compared to usual practice, and vice versa [44-46]. We plan to evaluate the clinical utility of the validated models by decision analysis. Decision analysis provides insight whether the model is better than usual care by combining test characteristics with evidence on consequences of the outcome, effects and burden of the further management, and costs [18]. In case a model is worth considering for implementation in clinical practice, it is necessary to determine the most optimal threshold value for risk classification. Finally, we will assess the effects of applying prediction models with tailored care paths on decision-making and health outcomes in Dutch obstetric care, as compared with care-as-usual (Expect Study II).

## Appendix

Multimedia Appendix 1 Search strategies first-trimester obstetric prediction models.

Multimedia Appendix 2 Data collection study questionnaires and medical records.

Multimedia Appendix 3 Reviewer comments (3 reviewers) and rebuttal grant application ZonMw. The Netherlands Organization for Health Research and Development, Pregnancy and Childbirth program (ZonMw grant 209020007). [ENGLISH].

## Abbreviations

AUROC: area under the receiver operating characteristic
GDM: gestational diabetes mellitus
LGA: large-for-gestational-age
MEC: medical ethical committee
PE: pre-eclampsia
PIH: pregnancy induced hypertension
PTB: preterm birth
SGA: small-for-gestational-age

## References

[1] Bonsel GJ, Birnie E, Denktas S, Poeran J, Steegers EAP (2010). Lijnen in de Perinatale Sterfte, Signalementstudie Zwangerschap en Geboorte 2010.

[2] van der Kooy J, Poeran J, de Graaf JP, Birnie E, Denktasş S, Steegers EA, Bonsel GJ (2011). Planned home compared with planned hospital births in the Netherlands: intrapartum and early neonatal death in low-risk pregnancies. Obstet Gynecol.

[3] Mol BWJ, Roberts CT, Thangaratinam S, Magee LA, de Groot CJM, Hofmeyr GJ (2016). Pre-eclampsia. Lancet.

[4] Koning SH, Hoogenberg K, Lutgers HL, van den Berg PP, Wolffenbuttel BH (2016). Gestational diabetes mellitus:current knowledge and unmet needs. J Diabetes.

[5] Araujo Júnior E, Peixoto AB, Zamarian ACP, Elito Júnior J, Tonni G (2017). Macrosomia. Best Pract Res Clin Obstet Gynaecol.

[6] Kleinrouweler CE, Cheong-See FM, Collins GS, Kwee A, Thangaratinam S, Khan KS, Mol BW, Pajkrt E, Moons KG, Schuit E (2016). Prognostic models in obstetrics: available, but far from applicable. Am J Obstet Gynecol.

[7] Al-Rubaie Z, Askie LM, Ray JG, Hudson HM, Lord SJ (2016). The performance of risk prediction models for pre-eclampsia using routinely collected maternal characteristics and comparison with models that include specialised tests and with clinical guideline decision rules: a systematic review. BJOG.

[8] Herraiz I, Arbués J, Camaño I, Gómez-Montes E, Grañeras A, Galindo A (2009). Application of a first-trimester prediction model for pre-eclampsia based on uterine arteries and maternal history in high-risk pregnancies. Prenat Diagn.

[9] Farina A, Rapacchia G, Freni Sterrantino A, Pula G, Morano D, Rizzo N (2011). Prospective evaluation of ultrasound and biochemical-based multivariable models for the prediction of late pre-eclampsia. Prenat Diagn.

[10] Park FJ, Leung CH, Poon LC, Williams PF, Rothwell SJ, Hyett JA (2013). Clinical evaluation of a first trimester algorithm predicting the risk of hypertensive disease of pregnancy. Aust N Z J Obstet Gynaecol.

[11] Skråstad RB, Hov GG, Blaas HK, Romundstad PR, Salvesen KÅ (2015). Risk assessment for preeclampsia in nulliparous women at 11-13 weeks gestational age: prospective evaluation of two algorithms. BJOG.

[12] E Holanda Moura SB, Park F, Murthi P, Martins WP, Kane SC, Williams P, Hyett J, da Silva Costa F (2016). TNF-R1 as a first trimester marker for prediction of pre-eclampsia. J Matern Fetal Neonatal Med.

[13] van Leeuwen M, Opmeer BC, Zweers EJK, van Ballegooie E, ter Brugge HG, de Valk HW, Visser GH, Mol BW (2009). External validation of a clinical scoring system for the risk of gestational diabetes mellitus. Diabetes Res Clin Pract.

[14] Thériault S, Forest J, Massé J, Giguère Y (2014). Validation of early risk-prediction models for gestational diabetes based on clinical characteristics. Diabetes Res Clin Pract.

[15] Syngelaki A, Pastides A, Kotecha R, Wright A, Akolekar R, Nicolaides KH (2015). First-trimester screening for gestational diabetes mellitus based on maternal characteristics and history. Fetal Diagn Ther.

[16] Lamain-de Ruiter M, Kwee A, Naaktgeboren CA, de Groot I, Evers IM, Groenendaal F, Hering YR, Huisjes AJ, Kirpestein C, Monincx WM, Siljee JE, Van 't Zelfde A, van Oirschot CM, Vankan-Buitelaar SA, Vonk MA, Wiegers TA, Zwart JJ, Franx A, Moons KG, Koster MP (2016). External validation of prognostic models to predict risk of gestational diabetes mellitus in one Dutch cohort: prospective multicentre cohort study. BMJ.

[17] Justice AC, Covinsky KE, Berlin JA (1999). Assessing the generalizability of prognostic information. Ann Intern Med.

[18] Steyerberg E (2009). Clinical Prediction Models a Practical Approach to Development, Validation, and Updating.

[19] Perined (2015). Perinatale Zorg in Nederland 2014.

[20] Nederlandse Vereniging voor Obstetrie en Gynaecologie (2014). Addendum bij multidisciplinaire richtlijn Hypertensieve aandoeningen in de zwangerschap uit 2011.

[21] Koninklijke Nederlandse Organisatie van Verloskundigen (2011). Standaard Hypertensieve aandoeningen tijdens de zwangerschap, bevalling en kraamperiode.

[22] Expect Studie.

[23] Molag ML, de Vries JH, Duif N, Ocké MC, Dagnelie PC, Goldbohm RA, van't Veer P (2010). Selecting informative food items for compiling food-frequency questionnaires: comparison of procedures. Br J Nutr.

[24] Bergink V, Kooistra L, Lambregtse-van den Berg MP, Wijnen H, Bunevicius R, van Baar A, Pop V (2011). Validation of the Edinburgh Depression Scale during pregnancy. J Psychosom Res.

[25] Bunevicius A, Kusminskas L, Pop VJ, Pedersen CA, Bunevicius R (2009). Screening for antenatal depression with the Edinburgh Depression Scale. J Psychosom Obstet Gynaecol.

[26] EuroQol Group (1990). EuroQol--a new facility for the measurement of health-related quality of life. Health Policy.

[27] Krabbe PF, Stouthard ME, Essink-Bot ML, Bonsel GJ (1999). The effect of adding a cognitive dimension to the EuroQol multiattribute health-status classification system. J Clin Epidemiol.

[28] van der Ploeg HM, Defares P, Spielberger CD (1980). Handleiding bij de Zelf-beoordelings Vragenlijst ZBV. Een nederlandstalige bewerking van de Spielberger State-Trait Anxiety Inventory.

[29] Marshall GN, Hays RD (1994). The patient satisfaction questionnaire short-form (PSQ-18).

[30] Truijens SE, Pommer AM, van Runnard Heimel PJ, Verhoeven CJ, Oei SG, Pop VJ (2014). Development of the Pregnancy and Childbirth Questionnaire (PCQ): evaluating quality of care as perceived by women who recently gave birth. Eur J Obstet Gynecol Reprod Biol.

[31] Nederlandse Vereniging voor Obstetrie en Gynaecologie (2005). Richtlijn Hypertensieve aandoeningen in de zwangerschap.

[32] Brown MA, Hague WM, Higgins J, Lowe S, McCowan L, Oats J, Peek MJ, Rowan JA, Walters BN, Austalasian Society of the Study of Hypertension in Pregnancy (2000). The detection, investigation and management of hypertension in pregnancy: full consensus statement. Aust N Z J Obstet Gynaecol.

[33] World Health Organization (1999). Definition, Diagnosis and Classification of Diabetes Mellitus and its Complications. Report of a WHO Consultation.

[34] Nederlandse Vereniging voor Obstetrie en Gynaecologie (2010). Richtlijn Diabetes Mellitus en Zwangerschap (2.0).

[35] Visser GHA, Eilers PHC, Elferink-Stinkens PM, Merkus HM, Wit JM (2009). New Dutch reference curves for birthweight by gestational age. Early Hum Dev.

[36] Vergouwe Y, Steyerberg EW, Eijkemans MJ, Habbema JD (2005). Substantial effective sample sizes were required for external validation studies of predictive logistic regression models. J Clin Epidemiol.

[37] van der Heijden GJ, Donders AR, Stijnen T, Moons KG (2006). Imputation of missing values is superior to complete case analysis and the missing-indicator method in multivariable diagnostic research: a clinical example. J Clin Epidemiol.

[38] Steyerberg EW, Vergouwe Y (2014). Towards better clinical prediction models: seven steps for development and an ABCD for validation. Eur Heart J.

[39] Debray TP, Vergouwe Y, Koffijberg H, Nieboer D, Steyerberg EW, Moons KG (2015). A new framework to enhance the interpretation of external validation studies of clinical prediction models. J Clin Epidemiol.

[40] Collins GS, Moons KG (2012). Comparing risk prediction models. BMJ.

[41] Moons KG, Kengne AP, Grobbee DE, Royston P, Vergouwe Y, Altman DG, Woodward M (2012). Risk prediction models: II. External validation, model updating, and impact assessment. Heart.

[42] Moons KG, Royston P, Vergouwe Y, Grobbee DE, Altman DG (2009). Prognosis and prognostic research: what, why, and how?. BMJ.

[43] van Gelder MM, Bretveld RW, Roeleveld N (2010). Web-based questionnaires: the future in epidemiology?. Am J Epidemiol.

[44] Altman DG, Royston P (2000). What do we mean by validating a prognostic model?. Stat Med.

[45] Moons KG, Altman DG, Vergouwe Y, Royston P (2009). Prognosis and prognostic research: application and impact of prognostic models in clinical practice. BMJ.

[46] Altman DG, Vergouwe Y, Royston P, Moons KG (2009). Prognosis and prognostic research: validating a prognostic model. BMJ.

