# Supplementary material for: External Validation Study of First Trimester Obstetric Prediction Models (Expect Study I): Research Protocol and Population Characteristics
Source: JMIR Res Protoc. 2017 Oct 26;6(10):e203. doi: 10.2196/resprot.7837 (PMC5680517; doi:10.2196/resprot.7837)
Supplement: Multimedia Appendix 1 [file resprot_v6i10e203_app1.pdf]

Pre-eclampsia: ("predictive model"[tiab] OR "predictive models"[tiab] OR prediction[tiab] OR "risk calculator"[tiab] OR "risk calculators"[tiab] OR "risk model"[tiab] OR "risk models"[tiab] OR "risk score"[tiab] OR algorithm*[tiab] OR "risk assessment"[tiab] OR nomogram[tiab] OR "prognostic model"[tiab] OR "prognostic models"[tiab] OR "scoring system"[tiab] OR "scoring systems"[tiab] OR "screening model"[tiab] OR "screening models"[tiab] OR "decision rule"[tiab] OR "decision rules"[tiab]) AND (preeclamp*[tiab] OR pre-eclamp*[tiab] OR "Pre-Eclampsia"[Mesh])

Gestational diabetes mellitus: ("predictive model"[tiab] OR "predictive models"[tiab] OR prediction[tiab] OR "risk calculator"[tiab] OR "risk calculators"[tiab] OR "risk model"[tiab] OR "risk models"[tiab] OR "risk score"[tiab] OR algorithm*[tiab] OR "risk assessment"[tiab] OR nomogram[tiab] OR "prognostic model"[tiab] OR "prognostic models"[tiab] OR "scoring system"[tiab] OR "scoring systems"[tiab] OR "screening model"[tiab] OR "screening models"[tiab] OR "decision rule"[tiab] OR "decision rules"[tiab]) AND ("gestational diabetes"[tiab] OR "pregnancy induced diabetes"[tiab] OR "pregnancy-induced diabetes"[tiab] OR "Diabetes, Gestational"[Mesh])

Spontaneous preterm birth: ("predictive model"[tiab] OR "predictive models"[tiab] OR prediction[tiab] OR "risk calculator"[tiab] OR "risk calculators"[tiab] OR "risk model"[tiab] OR "risk models"[tiab] OR "risk score"[tiab] OR algorithm*[tiab] OR "risk assessment"[tiab] OR nomogram[tiab] OR "prognostic model"[tiab] OR "prognostic models"[tiab] OR "scoring system"[tiab] OR "scoring systems"[tiab] OR "screening model"[tiab] OR "screening models"[tiab] OR "decision rule"[tiab] OR "decision rules"[tiab]) AND ("preterm labour"[tiab] OR "premature labour"[tiab] OR "premature labor"[tiab] OR "premature delivery"[tiab] OR "premature deliveries"[tiab] OR "premature parturition"[tiab] OR

"premature birth"[tiab] OR "preterm labor"[tiab] OR "preterm birth"[tiab] OR "preterm delivery"[tiab] OR "preterm deliveries"[tiab] OR "preterm parturition"[tiab] OR "Premature Birth"[Mesh])

Small-for-gestational-age: ("predictive model"[tiab] OR "predictive models"[tiab] OR prediction[tiab] OR "risk calculator"[tiab] OR "risk calculators"[tiab] OR "risk model"[tiab] OR "risk models"[tiab] OR "risk score"[tiab] OR algorithm*[tiab] OR "risk assessment"[tiab] OR nomogram[tiab] OR "prognostic model"[tiab] OR "prognostic models"[tiab] OR "scoring system"[tiab] OR "scoring systems"[tiab] OR "screening model"[tiab] OR "screening models"[tiab] OR "decision rule"[tiab] OR "decision rules"[tiab]) AND ("low birthweight"[tiab] OR "low birth weight"[tiab] OR "small for gestational"[tiab] OR "small for gestation"[tiab] OR "small-for-gestational"[tiab] OR "small-for-gestation"[tiab] OR "intrauterine growth restriction"[tiab] OR "intra-uterine growth restriction"[tiab] OR "intrauterine growth retardation"[tiab] OR "intra-uterine growth retardation"[tiab] OR "Infant, Small for Gestational Age"[Mesh])

Large-for-gestational-age: ("predictive model"[tiab] OR "predictive models"[tiab] OR prediction[tiab] OR "risk calculator"[tiab] OR "risk calculators"[tiab] OR "risk model"[tiab] OR "risk models"[tiab] OR "risk score"[tiab] OR algorithm*[tiab] OR "risk assessment"[tiab] OR nomogram[tiab] OR "prognostic model"[tiab] OR "prognostic models"[tiab] OR "scoring system"[tiab] OR "scoring systems"[tiab] OR "screening model"[tiab] OR "screening models"[tiab] OR "decision rule"[tiab] OR "decision rules"[tiab]) AND (macrosomi*[tiab] OR "large for gestational"[tiab] OR "large-for-gestational"[tiab] OR "high birthweight"[tiab] OR "high birth weight"[tiab] OR "large for gestation"[tiab] OR "large-for-gestation"[tiab] OR "Fetal Macrosomia"[Mesh])
