# Supplementary material for: External Validation Study of First Trimester Obstetric Prediction Models (Expect Study I): Research Protocol and Population Characteristics
Source: JMIR Res Protoc. 2017 Oct 26;6(10):e203. doi: 10.2196/resprot.7837 (PMC5680517; doi:10.2196/resprot.7837)
Supplement: Multimedia Appendix 2 [file resprot_v6i10e203_app2.pdf]

Table 1. Aspects covered by Pregnancy Questionnaire 1.

| Domain | Item | Sub-items |
|---|---|---|
| Sociodemographic characteristics | Ethnicity | Country of birth |
| | | Country of birth biological parents |
| | | Ethnic origin |
| | Highest level of education | |
| | Work | |
| | Volunteer work and informal care | |
| | Living situation | |
| Anthropometric data | Height | |
| | Pre-pregnancy weight | |
| Medical conditions | Chronic hypertension | |
| | Cardiovascular disease | Type |
| | Diabetes mellitus | Type |
| | Thromboembolism | |
| | Thyroid disease | Hypothyroidism<br>Hyperthyroidism |
| | Kidney disease | Type |
| | Autoimmune diseases | Systemic lupus erythematosus<br>Antiphospholipid syndrome |
| | Lung diseases | Type |
| | Gynaecologic history | Uterus anomaly |
| | | Cervical surgery |
| Obstetric history | Parity | |
| | Miscarriage | Date |
| | | Gestational age |
| | Termination | Date |
| | | Gestational age |
| | | Indication |
| | Ectopic pregnancy | Date |
| | | Gestational age |
| | Multiple birth | |
| | Pregnancy outcomes | Pre-eclampsia<br>HELLP syndrome<br>Gestational diabetes mellitus<br>Preterm birth<br>Placental abruption<br>Other |
| | | Management |
| | Labour and delivery | Mode of delivery |
| | | Hospital/home delivery |
| | Neonatal outcomes | Gender |
| | | Birth weight |
| | | Fetal mortality (antenatal, intrapartum) |
| | | Neonatal mortality |
| Lifestyle | Smoking | Status |
| | | Quantity |
| | Alcohol | Status |

| | | |
|---|---|---|
| | | Quantity |
| | Drugs | Status |
| | | Type(s) |
| | | Frequency |
| Medication | Name | Dose |
| | | Frequency |
| | | Duration |
| Vitamin and mineral supplements | Folic acid<br>Prenatal vitamins<br>General multivitamins<br>Vitamin D<br>Calcium<br>Iron | Brand name |
| | | Duration |
| | | Dose |
| | | Frequency |
| Dietary intake | Fruit intake | |
| | Selected items from Food Frequency Questionnaire tool:<br>Milk and buttermilk<br>Yoghurt and fromage frais<br>Yoghurt drinks and other dairy beverages<br>Chocolate milk<br>Custard and pudding<br>Dutch cheese<br>Non-Dutch cheese and cream cheese<br>Cheese spread<br>Bread spread<br>Cooking fat<br>Fish | Frequency |
| | | Average daily amount |
| Sun exposure | Skin type | |
| | Sun exposure | Week days |
| | | Weekend |
| | | Skin exposure |
| | | Sunscreen use |
| | Solarium | |
| Family history medical conditions (grandparents, parents and siblings) | Hypertension | |
| | Heart disease | |
| | Diabetes mellitus | Type |
| Family history obstetric outcomes | Adverse obstetric outcomes (mother and sister(s)) | Pre-eclampsia<br>HELLP syndrome<br>Gestational diabetes mellitus<br>Preterm birth<br>Low birth weight infant<br>High birth weight infant |
| | Women’s birth weight | |
| | Women’s gestation at delivery | |
| Mental health | Mental disorder | Type |
| | | Treatment |
| | Edinburgh Depression Scale | 10-items |

| | | |
|---|---|---|
| Health status | EuroQol-5D-3L | Mobility |
| | | Self-care |
| | | Usual activities |
| | | Pain/discomfort |
| | | Anxiety/depression |
| | Cognitive dimension | |
| | Evaluation overall health status | Visual analogue scale |
| Current pregnancy | Due date | |
| | Conception | Method of conception |
| | | Time to conception |
| | Caregiver (primary care/secondary care) | |
| | Multiple pregnancy | |
| | Vaginal bleeding | Duration |
| | | Quantity |
| Blood pressure measurement | Systolic and diastolic blood pressure | Measurement |
| | | Date of measurement |
| Heart rate measurement | Heart rate | Measurement |
| | | Date of measurement |

Table 2. Aspects covered by Postpartum Questionnaire 1.

| Domain | Item | Sub-items |
|---|---|---|
| Pregnancy outcome | Miscarriage | Gestational age |
| | Termination | Gestational age |
| | | Indication |
| | Ectopic pregnancy | Gestational age |
| | Delivery | |
| Pregnancy complications | Pregnancy induced hypertension | Gestational age |
| | | Treatment |
| | Pre-eclampsia | Gestational age |
| | | Treatment |
| | | HELLP syndrome |
| | Gestational diabetes mellitus | Gestational age |
| | | Treatment |
| | Threatened preterm labour | Preterm premature rupture of membranes |
| | | Admission |
| | | Treatment |
| | Growth disorders | Small-for-gestational-age infant |
| | | Low-for-gestational-age infant |
| | | Gestational age |
| Labour and delivery | Date | |
| | Gestational age | |
| | Hospital/home birth | |
| | Primary/secondary/tertiary care | Referral (indication) |
| | Mode of delivery | |
| | Assisted birth | |
| Neonatal outcomes | Multiple birth | |
| | Gender | |
| | Birth weight | |
| | Mortality | Antenatal |
| | | Intrapartum |
| | | Postpartum within 24 hours |
| | | Postpartum ≥24 hours |
| | Admission | Indication |
| | | Duration |
| | | Neonatal intensive care unit |
| | Congenital anomalies | |
| | Child's growth | Weight |
| | | Date measurement |
| Biological father | Ethnicity | Country of birth |
| | | Country of birth biological parents |
| | | Ethnic origin |
| | Anthropometric data | Length |
| | | Weight |
| | Father's birth weight | |
| | Father's gestation at delivery | |

Table 3. Aspects covered by Pregnancy Questionnaires 2, and 3 and Postpartum Questionnaire 2.

| Domain | Item | Sub-items |
|---|---|---|
| Pregnancy status (pregnancy questionnaire 2 and 3) | Pregnant | Gestational age |
| | Miscarriage | Gestational age |
| | Termination | Gestational age |
| | | Indication |
| | Ectopic pregnancy | Gestational age |
| | Gave birth | Date |
| | | Referral to postpartum questionnaire 1 and 2 |
| Maternal health status | EuroQol-5D-3L | Mobility |
| | | Self-care |
| | | Usual activities |
| | | Pain/discomfort |
| | | Anxiety/depression |
| | Cognitive dimension | |
| | Evaluation overall health status | Visual analogue scale |
| Patient satisfaction | Patient Satisfaction Questionnaire Short Form (PSQ-18) | 18-items capturing the most important characteristics of services and providers that might influence patient satisfaction with care (pregnancy questionnaire 2 and 3). |
| | Pregnancy and Childbirth Questionnaire (PCQ) | Quality of care during pregnancy and delivery as perceived by woman who recently gave birth (postpartum questionnaire 2). |
| | Primary/secondary care | Referral |
| | | Information |
| | | Collaboration |
| | Appointments (pregnancy questionnaire 2 and 3) | |
| | Information (pregnancy questionnaire 2 and 3) | Health |
| | | Medical examination(s) |
| | | Treatment(s) |
| | Intended place of birth (postpartum questionnaire 2) | |
| | Overall grade care during pregnancy (pregnancy questionnaire 2 and 3) | |
| | Overall grade care during labour and delivery (postpartum questionnaire 2) | |
| Anxiety | State anxiety of the State-Trait Anxiety Inventory (pregnancy questionnaire 2) | 20-items |
| | Concerns | |
| Costs of current pregnancy – | General practitioner | Consultation(s) (type and |

| | | |
|---|---|---|
| care for pregnant woman and their newborn(s) from the beginning of pregnancy up to ±6 weeks after the due date | Emergency department | frequency) |
| | Midwifery care | |
| | Obstetrician | |
| | Other medical specialist(s) | |
| | Other caregivers (e.g. dietician, physiotherapist) | |
| | Home care (domestic help and nursing) | |
| | Well-baby clinic (postpartum questionnaire 2) | |
| | Fetal ultrasound(s) | Type and frequency |
| | Admission(s) | Indication |
| | | Duration |
| | | Department |
| | Maternity care (postpartum questionnaire 2) | Duration |
| | Medication | Name |
| | | Dose |
| | | Duration |
| | | Frequency |
| | Over-the-counter medicines and vitamins/supplements | Name |
| | | Duration |
| | | Frequency |
| | | Cost |
| | Medical aid | Type |
| | | Cost |

Table 4. Data extracted from medical records and letters of discharge.

| **Domain** | **Item** | **Sub-items** |
|---|---|---|
| Obstetric history | Gravidity | |
| | Parity | |
| Caregiver | Primary/secondary/tertiary care | |
| | Referral | Date |
| | | Indication |
| Current pregnancy | Due date | |
| | Pregnancy outcome | Miscarriage |
| | | Termination |
| | | Ectopic pregnancy |
| | | Gave birth |
| | Method of conception | |
| | Medical indication secondary care/tertiary care | |
| | Admission | Date |
| | | Indication |
| | Highest diastolic blood pressure | |
| | Proteinuria | |
| | Complication (letter of discharge) | Hypertensive disorder |
| | | Gestational diabetes mellitus |
| Labour and delivery | Onset of labour | |
| | Mode of delivery | |
| | Assisted birth | |
| | Hospital/home delivery | |
| | Pain medication | |
| | Birth placenta | |
| | Amount of blood loss | |
| | Admission | Indication |
| | | Duration |
| Neonatal outcomes | Gestational age | |
| | Gender | |
| | Birth weight | |
| | Apgar score 1, 5, and 10 minutes | |
| | pH umbilical cord | Arterial |
| | | Venous |
| | Congenital anomalies | |
| | Paediatric consultation | |
| | Admission | Department |
| | Mortality | Date |
