# Supplementary material for: External Validation Study of First Trimester Obstetric Prediction Models (Expect Study I): Research Protocol and Population Characteristics
Source: JMIR Res Protoc. 2017 Oct 26;6(10):e203. doi: 10.2196/resprot.7837 (PMC5680517; doi:10.2196/resprot.7837)
Supplement: Multimedia Appendix 3 [file resprot_v6i10e203_app3.pdf]

ENGLISH

Reviewer comments (3 reviewers) Grant application ZonMw + Rebuttal

Subsidieprogramma / Subsidy programme	:	**Zwangerschap en geboorte**
Dossiernummer / Dossier number	:	**50-50200-98-063**
Aanvrager / applicant	:	**Prof. dr. M.E.A. Spaanderman**
Projecttitel / Project title	:	**External validity and impact of first-trimester obstetric prediction rules in the Netherlands**
Beoordelingscode / Assessment code	:	**R.2012.524**

1. Criteria

Legenda: VG (Very good), G (Good), S (Sufficient), M (Moderate), U (Unsatisfactory)

1.1 Objective, problem definition and assignment

VG	G	S	M	U
		X		

Consider the following factors:

- how clear and specific the objective is;
- how clear and verifiable the problem definition/assignment is and whether it is consistent with the objective;
- the value added to existing knowledge or practice;
- the theoretical or empirical evidence presented in support of the problem definition/assignment;
- the evidence presented to substantiate the value of the innovation or change in practice;
- the extend to which the desired practical change is specified.

This is a 2 part proposal which aims first to assess the validity of several first trimester obstetrical prediction rules in a Dutch population. The second aim is to develop and test interventions in those deemed to be at high risk. The methods are generally well described, and I believe that the investigative team is good. The analytical approach is well defined.
I have 2 major concerns however:
1. The test characteristics of most first trimester prediction rules are generally believe not to be good enough (ie both sensitive and specific enough)to use clinically, especially given the low prevalence for many outcomes. More thought and explanation should be given to this key issue.
2. The author's acknowledge that implementing a prediction rule only is useful if there are interventions to use that would decrease some of the adverse health outcomes. I dont know of any intervention or care path that will decrease the risk of preterm birth, preeclampsia, SGA or GDM. So I believe that these care paths would need to be developed and justified before starting this study. I doubt that the can even be developed, given the lack of evidence for most tested interventions.

1.2 Strategy

VG	G	S	M	U
		X		

Consider the following factors:

- clarity;
- adequacy in terms of problem definition/assignment;
- adequacy of chosen methods and analyses;
- if there is a target group:
    - the way in which the strategy reflects the factors gender, age, ethnicity and/or other characteristics relevant to the objective;
    - degree of collaboration with intermediate and/or ultimate target group (the patient/client perspective).

  With a implementation project:

- analysis of the context in which implementation is to take place;
- to which target groups are mentioned;
- a good mix of implementation activities (see notes);
- analysis of factors facilitating or hampering those activities;
- participation of stakeholders;
- prospect of structural incorporation in system;
- adequacy of process and effect evaluation design.

My main comments are on the last pages. Overall I felt like the population was well described and generalizable, and that the overall strategy was logical and well described. Given the structure of the Dutch system, I it seemed that collaboration was necessary.

My major strategy issues is listed on the prior page- what are the specific care paths and what evidence is there that any components are beneficial. Without this information, it is very difficult to judge this proposal.

1.3 Project group

VG	G	S	M	U
X				

Consider the following factors:
- relevant expertise;
- familiarity with area in question;
- prior activities and products.

A strength of this proposal is the research group. All relevant disciplines are represented.

1.4 Feasibility

VG	G	S	M	U
	X			

Consider the following factors:
- will it be possible to achieve the objective(s) using this strategy?
- availability of facilities/staff;
- realistic phasing and timetable.

I believe that the timeline for a study such as this is feasible, and that the sample size needed could be enrolled.

1.5 Overall quality assessment

VG	G	S	M	U
			X	

While this is an excellent investigative team studying an important topic, I am not enthusiastic that this study will meet it aims. In my opinion, the fatal flaw is that "care pathways" need to be developed to reduce adverse health outcomes in those who screen positive (from prediction rules). Unfortunately, there are virtually no interventions that have been documented in clinical trials to improve pregnancy outcomes for the conditions of interest in this study. For this reason, I am not enthusiastic about this study.

2. Budget

Legenda: TH (Too high), R (realistic), TL (too low)

2.1 Budget

TH	R	TL
	X	

I do nto see any significant issues with the budget.

Subsidieprogramma / Subsidy programme	:	**Zwangerschap en geboorte**
Dossiernummer / Dossier number	:	**50-50200-98-063**
Aanvrager / applicant	:	**Prof. dr. M.E.A. Spaanderman**
Projecttitel / Project title	:	**External validity and impact of first-trimester obstetric prediction rules in the Netherlands**
Beoordelingscode / Assessment code	:	**R.2012.525**

1. Criteria

Legenda: VG (Very good), G (Good), S (Sufficient), M (Moderate), U (Unsatisfactory)

1.1 Objective, problem definition and assignment

VG	G	S	M	U
				X

Consider the following factors:

- how clear and specific the objective is;
- how clear and verifiable the problem definition/assignment is and whether it is consistent with the objective;
- the value added to existing knowledge or practice;
- the theoretical or empirical evidence presented in support of the problem definition/assignment;
- the evidence presented to substantiate the value of the innovation or change in practice;
- the extend to which the desired practical change is specified.

It is unclear which models for predicting rules will be used.Eleven articles on predicting rules are listed but it is uncertain whether more will be added.Will the predicting value of each article be assessed selarately or will rules be selected from the articles. Will SGA, PTB, GDM, and PET studied independently? (Prefer PE for abbreviation as the "T" refers to toxaemia which is a historical feature of the disease.)It is difficult to see how a prediction model for HELLP syndrome could be found in the first trimester as mentioned in the model selection phase as this is a later complication of PE. It would be easier to assess an application if a clear indication of selected models is firstly given.

1.2 Strategy

VG	G	S	M	U
			X	

Consider the following factors:

- clarity;
- adequacy in terms of problem definition/assignment;
- adequacy of chosen methods and analyses;
- if there is a target group:
  - the way in which the strategy reflects the factors gender, age, ethnicity and/or other characteristics relevant to the objective;
  - degree of collaboration with intermediate and/or ultimate target group (the patient/client perspective).

With a implementation project:

- analysis of the context in which implementation is to take place;
- to which target groups are mentioned;
- a good mix of implementation activities (see notes);
- analysis of factors facilitating or hampering those activities;
- participation of stakeholders;
- prospect of structural incorporation in system;
- adequacy of process and effect evaluation design.

It is uncertain how many participants will have access to the internet as limited access will reduce the participation of minority groups or mothers from poor socioeconomic background, where the perinatal mortality rate is most likely the highest.Restricting the study internet users only could introduce bias to the study.

1.3 Project group

VG	G	S	M	U
	X			

Consider the following factors:

- relevant expertise;
- familiarity with area in question;
- prior activities and products.

The study consists of an experienced team of researchers from many disciplines and communities.

1.4 Feasibility

VG	G	S	M	U
				X

Consider the following factors:
- will it be possible to achieve the objective(s) using this strategy?
- availability of facilities/staff;
- realistic phasing and timetable.

This part is difficult to assess as it is uncertain how many studies will be used to select prediction rules.

1.5 Overall quality assessment

VG	G	S	M	U
			X	

A The study consists of two parts, the validation study and the impact study. It is difficult to assess the impact study without clear knowledge of the outcome of the validation study.

2. Budget

Legenda: TH (Too high), R (realistic), TL (too low)

2.1 Budget

TH	R	TL
	X	

The budget seems to be realistic.

Subsidieprogramma / Subsidy programme	:	**Zwangerschap en geboorte**
Dossiernummer / Dossier number	:	**50-50200-98-063**
Aanvrager / applicant	:	**Prof. dr. M.E.A. Spaanderman**
Projecttitel / Project title	:	**External validity and impact of first-trimester obstetric prediction rules in the Netherlands**
Beoordelingscode / Assessment code	:	**R.2012.526**

1. Criteria

Legenda: VG (Very good), G (Good), S (Sufficient), M (Moderate), U (Unsatisfactory)

1.1 Objective, problem definition and assignment

VG	G	S	M	U
		X		

Consider the following factors:

- how clear and specific the objective is;
- how clear and verifiable the problem definition/assignment is and whether it is consistent with the objective;
- the value added to existing knowledge or practice;
- the theoretical or empirical evidence presented in support of the problem definition/assignment;
- the evidence presented to substantiate the value of the innovation or change in practice;
- the extend to which the desired practical change is specified.

The applicants have provided sufficient background information. Prediction of adverse pregnancy outcomes using first trimester prediction rules is a clinically important area. The need for validation and impact is explained well. The objectives are stated clearly.
I am not able to fathom, the accuracy of the existing prediction rules and would appreciate further information about them. Furthermore, there is no explanation on how the outcomes have been chosen for the validation part of the study. The composite outcome in the impact is mainly neonatal and insuffcient information is provided on why this is chosen, considering that the validation part mainly looks for maternal adverse outcomes. There is insufficient detail on how the individual components of the neonatal composite adverse outcome were chosen.

1.2 Strategy

VG	G	S	M	U
	X			

Consider the following factors:

- clarity;
- adequacy in terms of problem definition/assignment;
- adequacy of chosen methods and analyses;
- if there is a target group:
    - the way in which the strategy reflects the factors gender, age, ethnicity and/or other characteristics relevant to the objective;
    - degree of collaboration with intermediate and/or ultimate target group (the patient/client perspective).

  With a implementation project:

- analysis of the context in which implementation is to take place;
- to which target groups are mentioned;
- a good mix of implementation activities (see notes);
- analysis of factors facilitating or hampering those activities;
- participation of stakeholders;
- prospect of structural incorporation in system;
- adequacy of process and effect evaluation design.

The applicants have targeted all pregnant women and that is relavant to the proposed study.They have calculated appropriate sample sizes for the validation and impact components of the study. There is sufficient details to ensure that they will be able to recruit through their collaborative network.
As a rule, a prediction model will need to have not more than 10 variables if there are 100 events. More clarity is needed how they will achieve this, given the number of models thay are evaluating. It is not very clear about the strategies to improve the accuracy of the existing models.
One of the main issues with prediction models is the treatment paradox, where the treatment prevents the outcome that may have otherwise occured. The application does not provide any details on how this will be done. They have provided an argument on why the study design is not RCT, due to fear of contamination if individual

patients are randomised. Since the care to the women is individualised, it may be less of a problem. however, the design as it involves less resources and time than an RCT.
The first step of the study involves evaluation of the prediction rules. There is lack of explanation on how the literature search will be done and if there will be any restrictions according to language or setting.
The team has individuals with sufficient experience to carry this study.
I am concerned that the time requested is ambitious, given the 4 components involved.

1.3 Project group

VG	G	S	M	U
	X			

Consider the following factors:
- relevant expertise;
- familiarity with area in question;
- prior activities and products.

The project group has the right mix of individuals to acrry out teh study

1.4 Feasibility

VG	G	S	M	U
			X	

Consider the following factors:
- will it be possible to achieve the objective(s) using this strategy?
- availability of facilities/staff;
- realistic phasing and timetable.

The staffing, cost and expertise are feasible.
They may struggle to complete in the time frame specified.

1.5 Overall quality assessment

VG	G	S	M	U
	X			

Overall it is a good quality application.
Further details are needed on how the multiple outcomes and variables will be dealt with.
They may be need to consider longer time period.
It may be appropriate to suggest funding in two phases dependent on the results of the validation study.

2. Budget

Legenda: TH (Too high), R (realistic), TL (too low)

2.1 Budget

TH	R	TL
	X	

seems appropriate, however since it is mainly in Dutch, I am unable to comment in detail

Rebuttal Spaanderman et al: External validity and impact of first-trimester obstetric prediction rules in the Netherlands. 50-50200-98-063

Reviewer id R 2012 524

1.1 Objective problem definition and assignment

The reviewer is overall content but expresses two concerns.

1. One concern is that the "test characteristics of most first trimester rules are generally believed to be not good enough (ie both sensitive and specific enough) to use clinically, especially given the low prevalence for many outcomes".

Unfortunately, the reviewer does not produce any literature showing that this is a general belief, or what arguments it is based upon.

Furthermore, despite what the reviewer implies, sensitivity and specificity are not fixed features of a prediction rule; instead they depend on which cut-off point is chosen. Even if, at a certain cut-off, sensitivity and specificity are not both optimal, a prediction test can be very useful in clinical practice. For instance, for outcomes with a low prevalence of 5% such as those in our study, a predictive test with a specificity of 98% and a sensitivity of only 70% will still detect more than 2/3s of the women with the prospective outcome although only a limited fraction of the women (5%) are test positive; of the test positives about 65% will have the outcome (if not prevented).

2. A second concern of the reviewer is that he or she does not "know of any intervention or care path that will decrease the risk of preterm birth, preeclampsia, SGA or GDM".

We would like to refer to a paragraph under "Relevance" (next to last) where we mention a number of interventions that have been proven, mostly in RCTs, to either reduce either the *risk* of these adverse outcomes or the *severity* of morbidity associated with these outcomes. Although the net effect of each of these individual interventions may be restricted, we believe that their combined effect can lead to significant reductions in maternal and child morbidity and mortality. It is important to mention also that interventions can also be initiated earlier in comparison with the present situation in which the VIL (Verloskundige Indicatie Lijst) is used. Therefore better preventive effects can be obtained.

We do not agree that care paths should be developed before the start of the study, because the prediction rules we identified so far are promising and the ingredients for effective care paths are available. They can be developed during the 1st part of the study (validation study).

1.2 Strategy

The reviewer refers to point 2 above (available effective interventions). We have explained why we think that beneficial effects can be achieved. In addition, prediction based care paths can reduce health care costs as (usually more expensive) 2nd line care actions will be focused on specific risks while women can remain under supervision of a 1st line midwife for general follow-up.

1.3 Project group

Our project group indeed comprises all relevant disciplines.

1.4 Feasibility

Feasibility is good according to the reviewer.

1.5 Overall quality assessment

We have argued that beneficial effects can be achieved by use of combinations of effective interventions (see earlier). Therefore we think that judging overall quality as "M" is unjustified. That

rating is also surprising in view of the fact that the four previous component ratings range from S to VG, and the average would be G.

2.1 Budget

Is reasonable according to the reviewer.

Reviewer id R 2012 525

We have serious problems with the work of this reviewer. His or her comments give us the strong impression that the application was either not carefully read, misapprehended, or both. In our opinion, we think it would be very unfair to give this reviewer's assessment much weight in the overall judgment of the application. We feel that his or her handling of the application is out of balance with the dedication, time and thought given to it by our team. We hope that the below considerations will make this further clear.

1.1 Objective, problem definition and assignment

The reviewer asks: "Will the predicting value of each article be assessed selarately or will rules be selected from the articles". Articles usually have no predicting value; and we think it is obvious from the proposal text (e.g. Strategy, par. 1.1, Model selection phase) that models will be selected from articles.

Another question is: "Will SGA, PTB, GDM, and PET studied independently?" As shown under 1.1, Model selection phase, published prediction rules predict one outcome each and it would be very illogical to validate prediction rules for other purposes than for which they were developed. Under 1.3, Data analyses, first sentence, it can be read that each prediction rule will be evaluated separately.

The reviewer further states that "It is unclear which models for predicting rules will be used". We are not planning to predict rules with models. Furthermore, we have made clear reference (again under Strategy, par. 1.1, Model selection phase) to published models that are promising and which will be evaluated in the validation study. More prediction rules may be published in the meantime (NB, 5 were published in 2011 alone) and it would be unwise not to leave room for any additional prediction rules to be included in the validation study.

1.2 Strategy

The reviewer seems to be basing his or her judgment on only 1 argument, namely that "it is uncertain how many participants will have access to the internet". Internet penetration rate is high in the Netherlands: 90% for all ages (December 2011), and virtually 100% among women of childbearing age.

Although the question is valid, the reviewer's judgment (M) is however completely out of balance, since all other features of the strategy seem to be ignored. (Clarity, adequacy in terms of problem definition/assignment, adequacy of chosen methods and analyses, if there is a target group: the way in which the strategy reflects the factors gender, age, ethnicity and/or other characteristics relevant to the objective; degree of collaboration with intermediate and/or ultimate target group (the patient/client perspective).)

1.4 Feasibility

The reviewer rates this aspect as "unsatisfactory" because he or she thinks this part is "difficult to assess as it is uncertain how many studies will be used to select prediction rules". We do not understand why feasibility would be difficult to assess when the eventual number of prediction rules to be selected is still uncertain. The variables contained in the prediction models are required to be easily

collectable either via routine care or questionnaire, so the number of studies/prediction rules is not relevant for feasibility.

Here again the reviewer ignores other aspects that should be evaluated (possibility to achieve the objectives using this strategy, availability of facilities/staff, realistic phasing and timetable).

Therefore, the judgment does not indicate that the application was read carefully, and it is again out of balance.

Reviewer id R 2012 526

1.1 Objective, problem definition and assignment

In the appendix we will give more details about the prediction rules that we found to be promising at the time of the writing of the application. We have chosen the outcomes PTB, SGA, LGA, GDM, PE(T)/HELLP syndrome, and asphyxia as eligible outcomes for prediction rules to be evaluated in the validation study (1.1 model selection phase), because they are relatively prevalent and important contributors to maternal and neonatal morbidity and mortality. We do not agree with the reviewer that the eligible outcomes in the validation study are mainly maternal. All have a possible impact on child health, and 4 of the 6 outcomes are predominantly child outcomes (PTB, SGA, LGA, asphyxia).

The reviewer is right in stating that the composite outcome in the impact study is neonatal. We chose not to combine specifically maternal and neonatal components in the composite outcome as interpretation of values for such an outcome is problematic. Therefore we separated maternal from neonatal outcomes, and although the composite outcome was the one we based our sample size upon, we defined a number of important secondary outcomes focused on maternal health.

The component outcomes in the composite outcome were chosen in such a way that they all had a clear association with neonatal morbidity as well as mortality. The components are: perinatal death, asphyxia, NICU admission, SGA p2.3, very preterm birth. Each of these outcomes alone is not prevalent enough to be used as an individual primary outcome. We chose not to use SGA 10.0 or preterm birth (<37 wks) as their association with morbidity / mortality is weaker than SGA 2.3 and very preterm birth (<32 wks) and at the same time they would put to much weight into the composite outcome (prevalences of 9-10% and 7.7% instead of 2.2% and 1.5%, respectively).

1.2 Strategy

The reviewer is generally content with the strategy. He or she states that “As a rule, a prediction model will need to have not more than 10 variables if there are 100 events.” This applies to model development, not model validation. For model validation, a rule of 100 events and 100 non-events can be maintained, irrespective of the number of variables in the prediction rule (Vergouwe et al 2005).

We think that the “treatment paradox” is a non-issue here since, during the validation study, we will not disclose predicted probabilities, nor will we apply care paths that may influence outcome.

The reviewer wants more information on how the literature search will be done, and if there will be any restrictions with respect to language or setting. Literature search will be done in PubMed, first by use of broad (sensitive) search terms, and then, on the basis of a review of 100 titles, abstracts and associated MeSH terms the search will be made more specific so as to be able to exclude non-relevant articles in an automated matter. Language will be restricted to English, Dutch, German, French, and Spanish, and in principle no restrictions will be applied to setting, but all papers will be evaluated with respect to applicability on a general population.

We are not sure what the reviewer means with the “4 components” involved in the study.

1.3 Project group: good

1.4 Feasibility

Again it is unclear why the reviewer thinks that the time frame will be insufficient. In any case, we will confer with other groups (UMCU and AMC) if data collection can be combined so that this part of the study can be shortened.

1.5 Overall quality assessment

The reviewer rates the application as good quality. Multiple outcomes is indeed an issue in both the validation and the impact study. Data analysis and ranking of prediction rules (validation study) is described under 1.3. The main outcome variable of the impact study is the composite neonatal outcome as for this outcome we calculated sample size. All other outcomes (mentioned under 2.2.2) will however be analyzed (2.3) and addressed in separate papers where necessary. As the reviewer suggests, we suggest that funding of the first study is conditional on the outcome of the first.

2 Budget: considered appropriate

Appendix: Variables contained in promising prediction rules at the time of application (Feb. 2012)

Yu et al (2005, outcome: PET, AUC : 0.72) : PE in history, ethnic origin, previous term birth, smoking, age, BMI (extra model with sonographic findings)

Tan et al (2007, outcome : PTB, AUC: 0.73) : Age, education, marital status, parity, start prenatal care, smoking, weight gain per week, medical complication

Poon et al (2008, outcome: PET, AUC: 0.80) : MAP, ethnicity, BMI, family history, parity, age, smoking, gestational age

Poon et al (2009, outcome: PET): maternal racial origin, BMI, and personal or family history of PE, MAP (extra model with uterine artery PI, PAPP-A, and PlGF)

Van Leeuwen et al (2009, outcome: GDM, AUC: 0.77): BMI, ethnic origin, diabetes in the family, history of GDM

Herraiz et al (2009, outcome: PET, AUC: 0.74): Etnic origin, BMI, family history of PET, parity, previous PET (extra model with sonographic findings)

Beta et al (2011, outcome: PTB, AUC : 0.67): Age, prepregnancy weight, height, ethnic origin, smoking, assisted conception, parity, previous PTB

Poon et al (2011, outcome: SGA, AUC : 0.72): Weight, Height, Age, Parous, Smoking, Racial origin, Chronic hypertension, Diabetes, Assisted conception. (Extra model with Delta NT, PAPP-A, ß-hCG)

Savvidou et al (2011, outcome: GDM, AUC : 0.82) : Age, BMI, Gestational age at sampling, Smoking, Race, Parity, Assisted Conception, Previous GDM, family history (extra models including HDL cholesterol, t-PA)

Nanda et al (2011, outcome: GDM, AUC : 0.79): Maternal age, BMI, Racial origin, Parity, History of GDM (extra models met adiponectin, SHBG).

North et al (2011, outcome: PET, AUC: 0.71): Age, MAP, BMI, fam hist, woman's birth weight, vag bleeding, prev miscarriage, TTP, fruit intake, alcohol consumption, smoking (extra model with US findings)
